# Prospective Risk Assessment of Medicine Shortages in Europe and Israel: Findings and Implications

**DOI:** 10.3389/fphar.2020.00357

**Published:** 2020-03-26

**Authors:** Nenad Miljković, Brian Godman, Milena Kovačević, Piera Polidori, Leonidas Tzimis, Torsten Hoppe-Tichy, Marika Saar, Ioan Antofie, Laszlo Horvath, Thomas De Rijdt, Róbert György Vida, Elena Kkolou, David Preece, Biljana Tubić, Joan Peppard, Alicia Martinez, Cristina Garcia Yubero, Ratiba Haddad, Dragana Rajinac, Pavle Zelić, Helena Jenzer, Franci Tartar, Gunda Gitler, Martina Jeske, Michal Davidescu, Guillaume Beraud, Darija Kuruc-Poje, Kristine Sakstrup Haag, Hanne Fischer, Inese Sviestina, Gordana Ljubojević, Anne Markestad, Vesna Vujić-Aleksić, Lana Nežić, Anica Crkvenčić, Johanna Linnolahti, Bogdan Ašanin, Nataša Duborija-Kovačević, Tomasz Bochenek, Isabelle Huys, Branislava Miljković

**Affiliations:** ^1^Institute of Orthopaedic Surgery “Banjica”, University of Belgrade, Belgrade, Serbia; ^2^Division of Clinical Pharmacology, Karolinska University Hospital, Karolinska Institutet, Stockholm, Sweden; ^3^Strathclyde Institute of Pharmacy and Biomedical Sciences, Strathclyde University, Glasgow, United Kingdom; ^4^Department of Public Health and Management, School of Pharmacy, Sefako Makgatho Health Sciences University, Pretoria, South Africa; ^5^Department of Pharmacokinetics and Clinical Pharmacy, University of Belgrade, Belgrade, Serbia; ^6^Department of Clinical Pharmacy, IRCCS, ISMETT, Palermo, Italy; ^7^Hospital Pharmacy Department, Chania General Hospital, Crete, Greece; ^8^Pharmacy Department, Heidelberg University Hospital, Heidelberg, Germany; ^9^Pharmacy Department, Tartu University Hospital, Tartu, Estonia; ^10^Hospital Pharmacy Department, Spitalul Clinic C. F. Cluj-Napoca, Cluj-Napoca, Romania; ^11^Department of Pharmaceutical Surveillance and Economics, University of Debrecen, Debrecen, Hungary; ^12^Pharmacy Department, University Hospitals Leuven, UZ Herestraat, Leuven, Belgium; ^13^Department of Pharmaceutics and Central Clinical Pharmacy, Faculty of Pharmacy, University of Pécs, Pécs, Hungary; ^14^Hospital Pharmacy Department, The Cyprus Institute of Neurology and Genetics, Nicosia, Cyprus; ^15^Leeds Medicines Advisory Service, The Leeds Teaching Hospitals NHS Trust, St James's University Hospital, Leeds, United Kingdom; ^16^Sector for Medicinal Products, Agency for Medicines and Medical Devices of Bosnia and Herzegovina, Banja Luka, Bosnia and Herzegovina; ^17^Faculty of Medicine—Department of Pharmacy, University of Banja Luka, Banja Luka, Bosnia and Herzegovina; ^18^Hospital Pharmacy Department, Midland Regional Hospital, Tullamore, Ireland; ^19^Servicio de Farmacia, Hospital Universitario Infanta Sofía, Madrid, Spain; ^20^Hospital Pharmacy Department, Hôpital Antoine Béclère, Clamart, France; ^21^Hospital Pharmacy Department, Clinical Centre of Serbia, Belgrade, Serbia; ^22^International Cooperation and Public Relation Department, Medicines and Medical Device Agency of Serbia, Belgrade, Serbia; ^23^Health Division, Berner Fachhochschule Health Professions Ernährung und Diätetik, Bern, Switzerland; ^24^Department of Hospital Pharmacy, General Hospital Celje, Celje, Slovenia; ^25^Hospital Pharmacy Department, Apotheke der Barmherzigen Brüdere. U., Linz, Austria; ^26^Pharmacy Department, University Clinic—State Hospital of Innsbruck, Innsbruck, Austria; ^27^Faculty of Social Sciences, The Graduate School of Business Administration, Tel Aviv, Israel; ^28^Médecine Interne et Maladies Infectieuses, CHU de Poitiers, Poitiers, France; ^29^Department of Public Health, Université Droit et Santé Lille 2, Lille, France; ^30^Hospital Pharmacy Department, General hospital “dr. Tomislav Bardek”, Koprivnica, Croatia; ^31^Nomeco-Hospital&Customer Service, Nomeco Commercial Affairs, Copenhagen, Denmark; ^32^Strategic Procurement and Supply of Pharmaceuticals, Amgros I/S, Copenhagen, Denmark; ^33^Hospital Pharmacy Department, Children's Clinical University Hospital, Riga, Latvia; ^34^Department of Physical Medicine and Rehabilitation “Dr Miroslav Zotović”, Banja Luka, Bosnia and Herzegovina; ^35^National Center for Medicine Shortages in Hospitals, Oslo universitetssykehus HF, Nydalen, Norway; ^36^Certification Department, The Republic of Srpska Agency for Certification, Accreditation and Quality Improvement in Health Care, Banja Luka, Bosnia and Herzegovina; ^37^Department of Pharmacology, Toxicology and Clinical Pharmacology, Faculty of Medicine, University of Banja Luka, Banja Luka, Bosnia and Herzegovina; ^38^Community Pharmacy Department, Pharmacy “Biljana”, Banja Luka, Bosnia and Herzegovina; ^39^Division DEFO, Finnish Medicines Agency, Helsinki, Finland; ^40^Department of Surgery, Department of Medical Ethics, Medical Faculty of the University of Montenegro, Podgorica, Montenegro; ^41^Department of Pharmacology and Clinical Pharmacology, Medical Faculty of the University of Montenegro, Podgorica, Montenegro; ^42^Department of Drug Management, Faculty of Health Sciences, Jagiellonian University Medical College, Krakow, Poland; ^43^Department of Pharmaceutical and Pharmacological Sciences, KU Leuven, Leuven, Belgium

**Keywords:** medicine shortage, risk assessment, mitigation strategy, substitution, Europe

## Abstract

**Introduction:**

While medicine shortages are complex, their mitigation is more of a challenge. Prospective risk assessment as a means to mitigate possible shortages, has yet to be applied equally across healthcare settings. The aims of this study have been to: 1) gain insight into risk-prevention against possible medicine shortages among healthcare experts; 2) review existing strategies for minimizing patient-health risks through applied risk assessment; and 3) learn from experiences related to application in practice.

**Methodology:**

A semi-structured questionnaire focusing on medicine shortages was distributed electronically to members of the European Cooperation in Science and Technology (COST) Action 15105 (28 member countries) and to hospital pharmacists of the European Association of Hospital Pharmacists (EAHP) (including associated healthcare professionals). Their answers were subjected to both qualitative and quantitative analysis (Microsoft Office Excel 2010 and IBM SPSS Statistics^®^) with descriptive statistics based on the distribution of responses. Their proportional difference was tested by the chi-square test and Fisher's exact test for independence. Differences in the observed ordinal variables were tested by the Mann-Whitney or Kruskal-Wallis test. The qualitative data were tabulated and recombined with the quantitative data to observe, uncover and interpret meanings and patterns.

**Results:**

The participants (61.7%) are aware of the use of risk assessment procedures as a coping strategy for medicine shortages, and named the particular risk assessment procedure they are familiar with failure mode and effect analysis (FMEA) (26.4%), root cause analysis (RCA) (23.5%), the healthcare FMEA (HFMEA) (14.7%), and the hazard analysis and critical control point (HACCP) (14.7%). Only 29.4% report risk assessment as integrated into mitigation strategy protocols. Risk assessment is typically conducted within multidisciplinary teams (35.3%). Whereas 14.7% participants were aware of legislation stipulating risk assessment implementation in shortages, 88.2% claimed not to have reported their findings to their respective official institutions. 85.3% consider risk assessment a useful mitigation strategy.

**Conclusion:**

The study indicates a lack of systematically organized tools used to prospectively analyze clinical as well as operationalized risk stemming from medicine shortages in healthcare. There is also a lack of legal instruments and sufficient data confirming the necessity and usefulness of risk assessment in mitigating medicine shortages in Europe.

## Introduction

Shortages of medicines have been on the rise globally this century (Gray and Manasse, 2012; American Society of Health-System Pharmacists, 2014; The Society of Hospital Pharmacists of Australia, 2017; Root, 2018; Miljkovic et al., 2019; Videau et al., 2019). Due to their multifaceted nature, there are currently over 26 definitions of medicine shortages from manufacturers, wholesalers, regulators, and healthcare providers (De Weerdt et al., 2015; Fox and McLaughlin, 2018). Contributing factors to shortages stem from the unique nature of the drug market itself, such as insufficient manufacturing capacity, a shortage of active pharmaceutical ingredients, and restricted distribution/allocation. These are all outside of the control of the pharmacist. Nevertheless, pharmacy departments may effectively manage drug shortages by implementing defined strategies ahead of the occurrence of a shortage. (Kaakeh et al., 2011; Ventola, 2011; Goldsack et al., 2014; De Weerdt et al., 2017; Fox and McLaughlin, 2018).

Risks from medicine shortages range from the basic risk of not being able to provide a patient with the medicine to providing a non-appropriate substitute thereby introducing the complication of medication errors (Parenteral Drug Association (PDA), 2014; Fox and McLaughlin, 2018). It is necessary to distinguish several risks emerging from shortages: 1) its occurrence; 2) treatment unavailable to the patient; and 3) providing a substitute that may not fully suit the patient's clinical needs. However, all risks may be prospectively analyzed separately for each medicine, based on the history of shortages, type of a medicine, the patients affected, and available alternatives (Canadian Pharmacists Association, 2010).

Medicine shortages have a direct impact on patient outcomes through disrupting the continuity of patient care and potentially deteriorating treatment outcomes originating from timely scarcity of drug supply and substitution with a less safe and effective alternative and the potential for medication errors (Iyengar et al., 2016; De Weerdt et al., 2017; Rinaldi et al., 2017; France Assos Santé, 2018; Phuong et al., 2019). Consequently, risk management plays an important role in healthcare settings *via* minimizing the likelihood of identified risk-related consequences associated with drug shortages (The Australian Council on Healthcare Standards (ACHS), 2013; Iyengar et al., 2016; American Society of Health-System Pharmacists, 2018; Root, 2018).

As stated by Acosta et al. (2019), managing medicine shortages should not consist solely of creating systems designed to gather information on the shortages themselves but also proposing proactive solutions for ongoing and emerging shortages (Acosta et al., 2019). Consequently, risk assessment plays a crucial role in understanding and mapping all risks emerging from potential drug shortages in respective healthcare environments (Australian Government Department of Health, 2018).

A comprehensive risk-based approach aimed at preventing and managing shortages assists in tackling multiple aspects of medicine shortages (Parenteral Drug Association (PDA), 2014). These risks exist at the manufacturing and supply chain levels as well as in the healthcare system itself. Risk management enables proactive risk identification, assessment, and risk control emerging from shortages, thereby reducing harm to patients (Parenteral Drug Association (PDA), 2014). Triage of risks allows for shortages to be proactively managed (Parenteral Drug Association (PDA), 2014). It is carried out *via* categorizing a medicine's criticality based on its therapeutic use/indication, patient clinical needs, likelihood of occurrence, and availability of alternatives (Parenteral Drug Association (PDA), 2014). Triage itself assists in the assessment of risks emerging from shortages through proposing and prioritizing risk corrective/control measures (Parenteral Drug Association (PDA), 2014).

Risk assessment may be performed both retrospectively and prospectively. Through incidence reporting and root cause analysis (RCA) driving incidents are retrospectively and meticulously analyzed so that their causes may be prevented in the future. The method focuses on the event rather than the process within which an incident occurs (Chiozza and Ponzetti, 2009). Conversely, prospective risk assessment, such as failure mode and effect analysis (FMEA), healthcare failure and mode effect analysis (HFMEA), hazard analysis and critical control points (HACCP) serves to evaluate processes and the causes of potential failures/risks/hazards, which may occur, to prioritize and prevent them from occurrence (Bonnabry et al., 2005; Chiozza and Ponzetti, 2009).

Regardless of its prospective or retrospective nature, risk assessment provides for the ability to manage risks in a manner most suitable for each healthcare setting as well as providing applicable solutions for containing and reducing detected risks (Clarkson, 2010). The assessments should take into account the causes of shortages, their duration, healthcare-setting utilization patterns of the medicines affected, and available substitutes. Successfully mitigating shortages also requires clear channels of communication with other healthcare professionals, particularly for the risk assessment's output and mitigation plans (Root, 2018). Furthermore, implementing processes systematically applied throughout a set of tailored procedures is necessary and provides the context in which all potential risks are identified, analyzed, evaluated, assessed, monitored, and reviewed (The Australian Council on Healthcare Standards (ACHS), 2013).

The majority of Council of Europe countries require that the pharmaceutical industry or the marketing authorization holder (MAH) inform their respective organization/institution responsible if a medicine will be unavailable due to postponed commercialization, market withdrawal, change in reimbursement schemes, or any other cause that might lead to a shortage (Bochenek et al., 2017). Article 23a, 2nd paragraph of EU Directive 2001/83/EC and article 27a, 2nd paragraph of Directive 2001/82/EC both stipulate that the member country's respective authority must be informed a minimum of two months prior to the expected shortage (European Medicines Agency (EMA) 2019b).

In 2014, both the pharmaceutical industry and European health authorities under the auspices of European Medicine Agency (EMA) and Parenteral Drug Association (PDA) jointly proposed a set of activities to address medicine shortages that were not solely based on notification strategies (Parenteral Drug Association (PDA), 2014). They aimed to tackle shortages in a prospective manner *via* preventative actions and communication strategies oriented also to the production of medicines and their quality assessment (Parenteral Drug Association (PDA), 2014). The authors believe this to be the first time risk assessment has been proposed as a mitigation strategy. Similarly, an initiative proposed by the Institute for Safe Medication Practices (ISMP) Canada was also based on prospective risk assessment aimed at detecting risks of all possible shortages as well as reducing their potential impact on patient health *via* failure mode and effect analysis (FMEA) (Institute for Safe Medication Practices Canada, 2006). The concept of risk assessment is also present in the USA and Australian [Therapeutics Goods Administration (TGA)] guidelines, where the critical need of different medicines is assessed in terms of shortage impact analysis [(the American Society of Health System Pharmacists (ASHP)] and patient safety risks (TGA) (Australian Government Department of Health, 2018; Fox and McLaughlin, 2018).

Rodriguez-Gonzalez et al. note that prospective risk assessment is beneficial in regard to medication administration (Rodriguez-Gonzalez et al., 2015). Therefore, such assessment may also be extended into the risk due to medicine shortages. In view of the first-hand nature in which they have to deal with shortages, a detailed exploration of the experience of healthcare professionals, as well as other healthcare stakeholders, concerning risks is central to minimizing the impact of shortages. Specifically, which shortages pose the greatest risk to patient health and the potential solutions for their management. Consequently, the aims of this study were firstly to elucidate the concept of risk-prevention among healthcare experts; secondly, ascertain existing strategies for minimizing risks for patient health through risk assessment and their experiences with implementing them. Thirdly, use the findings to provide future directions.

## Methodology

A survey was conducted between July, 2018, and March, 2019. It was distributed *via* e-mail to members of the European Cooperation in Science and Technology, COST Action 15105, a research network of consisting of representatives from 28 member countries who are focused on research in medicine shortages (COST, 2015; COST, 2018). The survey was also provided to volunteering hospital pharmacists who are members of the European Association of Hospital Pharmacists (EAHP) and any of their associated colleagues (other healthcare professionals) who chose to fill it out.

The survey included a semi-structured questionnaire of 38 questions across three sections, covering: (i) concepts of risk assessment in healthcare settings; (ii) aspects of implementation of risk assessment in medicine substitution due to medicine shortages; and (iii) outputs of risk assessment conducted in order to change therapy due to shortages (**Supplementary Material**). The survey was initially piloted among five experienced hospital pharmacists from separate countries involved in both clinical practice and academia relating to issues of medicine shortages. As all participants are experienced healthcare professionals who can provide future direction, we have listed all participants as co-authors in a similar manner to Bochenek et al. (2017).

As to enhance their robustness, the survey findings' accuracy have been reviewed together with the co-authors. Study rigor was additionally assured by including available published scientific literature and legislation relevant to risk assessment as part of the overall data analysis.

Both qualitative and quantitative data analyses were performed following a case-oriented methodological framework in order to encompass all aspects of risk mitigation strategies applied (Yin, 2003). Тhe quantitative data analysis was conducted with Microsoft Office Excel 2010 and IBM SPSS Statistics^®^ using descriptive statistics based on the distribution of responses expressed in percentages. The percentage next to each rating in the survey results represents the percentage of total respondents that selected a respective answer. The difference in proportions was tested by the chi-square test and Fisher's exact test for independence, while differences in the observed ordinal variables were tested by the Mann-Whitney or Kruskal-Wallis test. The qualitative data were examined, categorized, tabulated and recombined with quantitative data in order to observe, reveal and interpret meanings and patterns, and construct conclusions.

No ethical approval, neither written informed consent from participants, were sought for this study as the information obtained *via* the survey was practice-base oriented and publicly available. In addition, the healthcare professionals freely participated and no patients were involved, as in line with similar such studies in this and related fields (Godman et al., 2014; Moon et al., 2014; Bochenek et al., 2017). Due to the subject matter not concerning patients nor private information, no institutional ethical approval was required by the authors' home countries nor called for by any specific guidelines.

Twenty-four countries that are members of the COST Action 15105 were included in this study, 20 from the European Union (EU) and 4 non-EU.

## Results

Overall, there were 34 responses from 24 countries (out of the then 28 total COST Action 15105 countries), response rate: 85.7% (**Table 1**).

**Table 1:** Participant background (region, occupation, number of hospital pharmacists by pharmacy and population).

	Number of respondents	%*
**Participants**	34	100
EU-countries	25	73.5
Non-EU countries	9	26.5
Europe		
Northern	5	14.7
Central	6	17.6
Western	4	11.7
South-eastern	19	55.8
**Occupation and participants’ countries**		
Hospital pharmacist (Austria, Bosnia and Herzegovina, Belgium, Croatia, Cyprus, Greece, Estonia, France, Germany, Hungary, Ireland, Latvia, Montenegro, Norway, Romania, Slovenia, Serbia, Switzerland)	23	67.6
Community pharmacist (Bosnia and Herzegovina; Montenegro)	2	5.8
Wholesaler pharmacist (Denmark)	2	5.8
Agency-regulatory pharmacist (Serbia, Finland, Bosnia and Herzegovina)	3	8.8
Medical doctor (Bosnia and Herzegovina, France)	3	8.8
Clinical system manager (Israel)	1	2.9
Number of hospital pharmacists per pharmacy in the participants’ country**		
< 5	22	64.7
5–9	7	20.6
> 10	5	14.7
Number of hospital pharmacists per 100,000 population in the survey respondents’ country**		
< 5	15	44.1
5–9	11	32.3
> 10	8	23.5

*Due to rounding, some totals may not correspond with the sum of the separate figures.

**Data was obtained via the country reports of spring 2018. The information is based on data included in the 2017 results for the EAHP Statement survey. (Horak et al., 2018)

The majority of the participants work as hospital pharmacists (67.6%—23 participants), while those representing the community/wholesaler pharmacists were lowest in proportion to the total number of all participants (5.8%—2 participants).

### Risk Assessment as a Medicine Shortage Mitigation Strategy

The findings here indicate that five European countries have defined acts addressing shortages, which require risk-assessment implementation should a medicine shortage occur. See **Table 2** for risk assessments embedded in the legislation by respective country including those where this is expected to happen in the near future.

**Table 2:** Participants’ answers according to risk assessment embedded in legislation on medicine shortages.

Country	Access to information on alternatives *via* national shortages databases*	Legislation act	Access
Belgium	Existing information on available alternatives, with additional details	Royal decree 1885 will be updated with the bill 55K229 on medicine shortages coping strategies including risk assessment by 31 January 2020	https://www.rtbf.be/info/belgique/detail_la-chambre-approuve-une-proposition-de-loi-contre-la-penurie-de-medicaments?id=10392783
Bosnia and Herzegovina	No data base available	Medicinal products and medical devices Act (“Official Gazette of B-H, no.58/08”) (Government of Bosnia and Herzegovina, 2008)	http://www.almbih.gov.ba/en/_doc/regulative/medicinal_products_and_medical_devices_act.pdf
Denmark	Existing information on available alternatives, with additional details	The Danish Medicine Agency regulation on reporting obligation for drug suppliers in Denmark (Danish Medicines Agency, 2018)	https://laegemiddelstyrelsen.dk/da/godkendelse/kontrol-og-inspektion/alvorlige-forsyningsvanskeligheder/#
France	No information provided	Décret n° 2016-993 du 20 juillet 2016 relatif à la lute contre les ruptures d'approvisionnement de medicaments (Le Gouvernement français, 2016)	https://www.legifrance.gouv.fr/affichTexte.do?cidTexte=JORFTEXT000032922434&categorieLien=id
Hungary	Existing information on available alternatives, without additional details	Act XCV of 2005 on Medicinal Products for Human Use and on the Amendment of Other Regulations Related to Medicinal Products (Government of Hungary, 2005)	https://net.jogtar.hu/jogszabaly?docid=a0500095.tv&dbnum=62&getdoc=1
Serbia	No data base available	Law on Medicines and Medical Devices (The Official Gazette of the Republic of Serbia, 2010)	https://www.alims.gov.rs/eng/regulations/law-on-medicines-and-medical-devices/
Switzerland	No information provided	The Swiss Federal Act on Medicinal Products and Medical Devices (Therapeutic Products Act) and its ordinances (The Federal Assembly of the Swiss Confederation, 2000)	https://www.admin.ch/opc/en/classified-compilation/20002716/201901010000/812.21.pdf

*According to EMA – European Medicines Agency or available literature for non-EU countries.

TPA, Therapeutic Products Act.

Generally for life saving medicines (i.e., antibiotics, immunoglobulins oncology medicines and vaccines^1^—see **Table 3**), national authorities perform risk assessment for medicines affected by shortages in Denmark, Finland, Germany, Hungary, and Italy. Irish and UK medicine shortage management guidelines that encompass risk assessments have been developed (Health Products Regulatory Authority (HPRA), 2018; Root, 2018). Switzerland initiated risk assessment 18 years ago in practice (the Swiss Federal Act on Medicinal Products and Medical Devices). Hungary has only recently implemented risk assessment (Government of Hungary, 2006)

**Table 3:** Medicines under risk assessment during shortages.

Medicine	Country of survey participant	(%)* of total
Antibiotics	France, Finland, Bosnia and Herzegovina, Belgium, UK, Ireland, Austria	20.6
Oncology medicines	Austria, Greece, Romania, Montenegro	11.8
IVIG	Austria, Belgium, Finland, France, Romania	14.7
Blood products (human albumin)	Austria, Belgium, Cyprus, Finland	11.8
Vaccines	Austria, Belgium, Finland, France	11.8
Insulins	Bosnia and Herzegovina, Finland	5.9
Oral anticoagulants	Serbia	2.9
Diuretics (furosemid)	Serbia	2.9
Nutritional liquids	Finland	2.9
Adrenalin injections	Finland	2.9
Antihypertensives	Finland	2.9
Anesthetics	Israel	2.9

*Percentage of those who use risk assessment sorted by highest.

IVIG, immunoglubulins.

Although Acosta et al., 2019 reported numerous publications addressing multiple aspects of shortages in Europe, including their mitigation (Acosta et al., 2019), our findings show that 22 (64.7%) of all survey participants were unaware of any published governmental or non-governmental documents on applied risk assessment dedicated to shortages in their respective country. Twenty-nine of 34 (85.3%) were also not informed on any impact assessment of a respective shortage-mitigation strategy (see **Table 4**).

**Table 4:** Risk assessment in medicine shortages.

Selected survey questions and answers	Number of responses	%
Are you aware of any risk assessment procedures that can be used to mitigate medicine shortages? (answered “YES”)	21	61.7
The type of risk assessment:		
FMEA	9	26.4
HFMEA	5	14.7
HACCP	5	14.7
RCA	8	23.5
Other	2	5.8
Where does risk assessment take place		
Hospital pharmacy	9	26.5
Community pharmacy	2	5.8
Wholesaler facility	3	8.8
Healthcare authority	2	5.8
Are you able to describe the steps of the risk assessment procedures you are familiar with? (answered “YES”)	18	52.9
Do you implement risk assessment in your daily work? (answered “YES”)	15	44.1
Are you conducting risk assessment within multidisciplinary teams? (answer “YES”)	12	35.3
The type of professions included in the multidisciplinary team:		
Physician	7	20.6
Pharmacist	6	17.6
Nurse	4	11.7
Quality control/procurement department	7	20.6
	**Answered “YES”, Number of responses**	**%**
Is there a defined act stipulating the implementation of risk assessment during medicine shortages embedded within legislation in your country?	5	14.7
Are you aware of any other legislation which is to be implemented in the near future in your country?	4	11.7
Are you able to say how long risk assessment related to medicine shortages has been implemented in your country?	8	23.5
Are you aware of any initiative for the implementation of risk assessment related to medicine shortages expected to take place in the near future in your country?	7	20.6
Are there any published, governmental or non-governmental documents on applied risk assessment in your country?	12	35.3
Are there any published governmental or non-governmental documents dedicated to studying ways to reduce the impact of medicine shortages? Specifically, any documents related to applied risk assessment measures as a mitigation strategy in your country?	5	14.7
Are risk assessment procedures part of the medicine shortage protocols/mitigation strategy in your workplace?	10	29.4
When conducted, are you obligated to report on the results of the risk assessment to official institutions inside your country?	4	11.8
When completed, do you share the outcomes of the performed risk assessment for a specific medicine with other stakeholders in your country (such as manufacturers, health authorities, national/private health-insurance funds)?	6	17.6
Do you perform risk assessment together with other medicine shortage mitigation strategies?	7	20.6
According to your professional experience, have you been able to mitigate medicine shortages successfully with risk assessment procedures?	15	44.1
Do you keep records on how quickly you can manage risk assessment when a medicine shortage abruptly occurs?	3	8.8
Do you find risk assessment useful as a medicine shortage mitigation strategy?	29	85.3

Due to rounding, some totals may not correspond with the sum of the separate figures.

FMEA, Failure Mode and Effect Analysis; HFMEA, Healthcare Failure Mode and Effect Analysis; HACCP, Hazard Analysis and Critical Control Points; RCA, root cause analysis.

The participants (61.7%) are aware of the use of risk assessment procedures as a coping strategy for medicine shortages (**Table 4**). Twenty-seven participants named the particular risk assessment procedure they are familiar with: 26.4%—the FMEA, 23.5%—RCA, 14.7%—the HFMEA and 14.7%—the HACCP (**Table 4**). 52.9% described risk-assessment steps. The participants from Bosnia and Herzegovina, Denmark, Ireland, and Switzerland noted an approach more oriented to a retrospective risk analysis. Finnish and French participants report identifying root causes; analyzing risks and prioritizing needs, proposing alternatives according to recommendations. The latter do so according to the Agence Nationale de sécurité du Médicament et des produits de santé (ANSM) and The Haute Autorité de santé (HAS). 44.1% implement risk assessment in their daily work. Multidisciplinary teams (35.3%) consisting of physicians, pharmacists, nurses, and quality/procurement office members (20.6%; 17.6%; 11.7%; and 20.6%, respectively) conduct assessments (**Table 4**). Answers from Austria, Bosnian and Herzegovina, Denmark, and Finland reveal that such teams may have different members depending on the assessment; such as reconstitution of medicines, cross-departmental processes, quality control, logistics, and supply chain.

### The Structure and Process of Risk Assessment Procedures *via* Triage

Risk triage in practice is not universal. Whereas 10/34 participants from seven countries (29.4%) stated they apply risk assessment procedures in everyday work, 24 participants (70.6%) from 13 countries do not consider risk assessment to be currently integrated into their work to establish such strategies.^2^ Doing so requires a significant amount of time. This is born out in the findings where actively working on shortage mitigation accounts for slightly more than 10% of full-time equivalents (FTE) in Belgium, while it is approximately 5% in Finland and 30% in Norway. For those having risk assessment procedures in place, their update is conducted quarterly in France, Hungary, Switzerland, and the UK, monthly in Norway, annually in Bosnia and Herzegovina, and every 2 to 3 years in Finland.

The participants do recognize a variety of risks where medicine substitution is an important risk in eleven countries (**Table 5**). These risks also include insufficient single-dose barcode packs, available medicine in non-uniform concentrations, look-alike/sound-alike (LASA) medicines, unknown preparation, and administration methods as well as therapy duplication.

**Table 5:** Risks detected while conducting risk assessment in medicine shortages.

Country	Risks
Belgium, Bosnia and Herzegovina, France, Germany, Italy, Montenegro, Norway, Romania, Serbia, Spain, the UK	Risk due to medicine substitution/different usage of alternative medicine/difference in alternative's administration patterns
Bosnia and Herzegovina	Risk from medication errors due to substitution
Bosnia and Herzegovina	Risk from unforeseen adverse events due to substitution
Ireland	Risk from miss-communication among stakeholders due to shortages
Belgium	Risk from not having single barcodes packs due to shortages
Belgium	Risk from look-alike/sound-alike medicines in substitution due to shortages
Ireland, Finland, Norway, Spain, Romania	Risk for continuous medicine supply interruption in shortages
Switzerland	Risk from depending on raw material providers situated in Asian countries in shortages
France	Risk of having to prioritize patients when it comes to dispensing medicines due to shortages

Moreover, MAHs are legally required to report a potential shortage and its related risks to the health authorities (European Medicines Agency (EMA) 2019b). In contrast, Bochenek states that healthcare professionals implement reporting shortages heterogeneously, which may even be done *via* voluntary, informal networks but is not carried out by regulation (Bochenek et al., 2017). This informal nature is also conspicuous in our findings where there is an absence of reporting risk-assessment results to government authorities, with 30/34 participants (88.2%) not reporting their findings to their respective official institutions while four participants from Finland, Montenegro, Norway, and Romania, (11.8%) do so (**Table 4**).

### The Implications of Risk Assessment Procedures Regarding the Medicine Shortage Mitigation Process

Seven of 34 (20.6%) participants apply risk assessment in conjunction with increased stocks of medicines (held by the manufacturer, wholesaler, or pharmacy) to respond to shortages. Finland's Act on Mandatory Reserve Supplies requires that predetermined groups of medicines based on risk assessment are reviewed on a 2-year basis and that safety stocks are in place (The Finish Medicines Agency (FIMEA), 2008).

Twenty-nine of 34 (85.3%) participants consider risk assessment to be a useful mitigation strategy and 15/34 (44.1%) report to mitigate shortages successfully *via* risk assessment (see **Table 4**). As presented in **Table 6**, participants of eight countries consider strategies to be successful only if patient harm is prevented in shortages. They also view strategies as successful if: 1) they are able to compound medicine affected by a shortage; and 2) they are able to prioritize patients based on their clinical status to receive treatment (**Table 6**).

**Table 6:** What is considered to be a mitigation success in shortages among participants.

“What is a success in mitigating shortages?”	Country
*“When we don't have to let patients untreated.”*	Germany
*“No obvious harm to patients.”; “The shortage was managed without patient harm.”; “Preventing harm to patients through overcoming complete medicine shortages and finding alternatives.”*	Bosnia and Herzegovina, Ireland, Serbia, UK
*“Shortages were not met by healthcare professionals and patients.”*	Finland
*“The number of reported shortages in Norway is growing rapidly but the extra cost is reduced. The time spent at each hospital handling shortage is not growing proportionally to the increase in reported shortages.”*	Norway
*“We managed to get necessary medicines.”*	Slovenia
*“Keeping the medicine for vulnerable population-those who cannot be treated with alternative.”*	Israel
	Switzerland
*“Efficient prioritization of patients and effective communication with a physician”*	France
*“Rational use of product severely affected by a shortage or providing a suitable alternative”*	Belgium
*“Being able to redistribute a medicine, compound or import it.”; “Having tools for: 1. Communicating if a product is in shortage to the prescribers and nurses 2. Drug shortage catalogue and reporting systems (institutional and national) 3. Finding alternative sources (e.g.: importation) 4. Change in formulary”*	Hungary

When shortages abruptly occur, only 3/34 (8.8%) participants stated that they do keep records on how quickly they manage risk assessment (**Table 4**). In Norway, it takes up to 24 h to assess risk and provide guidelines on antibiotic substitution in cooperation with their National Medicine Agency. Moreover, Norway's National Centre for Medicine Shortages in Hospitals reportedly performed more than 100 risk assessments in the year preceding the survey. In addition, hospital pharmacists routinely conduct formal practice-based assessments in every day practice in Austria, Germany, and Hungary, even though there are no formal risk assessment procedures for shortages in place.

There are three factors among participants determining the number of assessments conducted and procedures used in the year preceding the survey: 1) a higher number of multidisciplinary members on the team; 2) the participant being aware of risk assessment; and 3) the participant implementing it in everyday practice (p < 0.005) (**Table 7**). No geographical difference was observed concerning the application of risk assessment, including the density of hospital pharmacists in the participants' countries (p > 0.005) (**Table 7**).

**Table 7:** Differences in observed ordinal variables.

Participants by geographical region; density of hospital pharmacists; survey questions regarding the implementation of risk assessment	The number of risk assessments performed in the year preceding the survey			The number of different risk assessment procedures used by the participants		
	Mean rank	Median [IQR]	p-value*	Mean rank	Median [IQR]	p-value*
**European region**						
Northern	16.67	50 [25–75]	0.374	14.25	1 [0.5–1.5]	0.634
Central	8.38	0[0–2]		15.25	0.5 [0–1]	
Western	13.67	5 [2.5–5.5]		18.88	1 [0.5–1]	
Southeastern	11.65	1 [0–4]		19.03	1 [0–2]	
**Number of hospital pharmacists per 100 000 of the population**						
<5	10.60	0 [0–4]	0.478	19.43	1 [0–2]	0.356
5–9	10.71	1 [0–2.5]		15.48	1 [0–1]	
>10	14.40	5 [0–50]		14.71	1 [0–1]	
**Number of hospital pharmacists per pharmacy**						
< 5	10.10	1 [0–13.5]	0.281	16.02	1 [0–1.5]	0.661
5–9	14.88	4.5 [2–27.5]		19.50	1 [0.5–1]	
>10	14	3 [1.5–51.5]		17.60	1 [1–1.5]	
**Number of professionals in the risk assessment team**						
0	8.36	0 [0–1]	0.012	16.26	0 [0.5–1]	0.340
1	15.33	6 [3–33]		17.38	1 [0.5–1.5]	
3	21	55 [10–100]		22.50	1 [1–1]	
4	16.67	5 [0.5–8.5]		24.63	1 [1–1.5]	
**Are you aware of any risk assessment procedures that can be used to mitigate medicine shortages?**						
No	9.64	0 [0–2]	0.278	8	0 [0–0]	< 0.001
yes	13.03	2.5 [0–8]		23.38	1 [1–2]	
**Do you implement risk assessment in your daily work?**						
No	7.96	0 [0–0.5]	0.002	14.95	0 [0–1.5]	0.048
Yes	16.41	6 [3–31]		20.73	1 [1–1]	
**Are risk assessment procedures part of the medicine shortage protocols/mitigation strategy in you workplace?**						
No	8.63	0 [0–0.5]	< 0.001	15.28	0 [0–1.5]	0.123
Yes	18.31	8 [4.5–31]		20.95	1 [1–1]	
**Do you perform risk assessment together with other medicine shortage-mitigation strategies?**						
No	10.17	0 [0–4]	0.012	16.54	1 [0–1]	0.620
Yes	18.60	12 [5–50]		18.71	1 [1–1]	

*p-value estimated by Mann-Whitney or Kruskal-Wallis test, when appropriate.

Mean rank assigned to ordinal variables corresponding to the participants' characteristics and their answer.

IQR, interquartile range.

## Discussion

All causes driving medicine shortages bare significant risks to patient treatment (Birgli, 2013; Fox and McLaughlin, 2018). Manufacturing problems are difficult to predict and account for, both locally and globally, particularly when a single manufacturer produces medicines for several MAHs (Health Products Regulatory Authority (HPRA), 2018). For instance, there has been a recent case indicating the potential human carcinogenic impurity of N-nitrosodimethylamine (NDMA) found in valsartan and ranitidine (Food and Drug Administration (FDA), 2019; Food and Drug Administration (FDA), 2020). This finding provoked unexpected shortages and showed how healthcare professionals should be better informed in order to mitigate them (Government of the United Kingdom, 2018; Government of the United Kingdom Department of Health and Social Care, 2019b). Expected discontinuation in production, limited capacity, and pharmaceutical industry consolidation, however, are more predictable and, therefore, manageable (Birgli, 2013).

Regardless of predictability, it is still necessary to be prepared for shortages and to assess a variety of risks stemming from them. Interruptions in medicine production and their supply chains may occur due to a number of factors (quality, legal, economic, or market). Moreover, there are substantial clinical and safety risks when patients do not receive treatment or appropriate substitutes (Parenteral Drug Association (PDA), 2014). These risks need to be addressed in a proactive manner in order to reduce harm, assure patient safety and optimize health outcomes. To the best of our knowledge, we believe this is the first paper to address the application of prospective risk assessment in medicine shortages in healthcare across Europe.

Currently, the latest guidelines on managing shortages proposed by the ASHP, the TGA, and the EMA are based on risk assessment involving both manufacturing and distribution processes, as well as possible risks to patients when introducing therapeutic alternatives (Australian Government Department of Health, 2018; Fox and McLaughlin, 2018; European Medicines Agency (EMA) 2019a; European Medicines Agency (EMA) 2019b; European Medicines Agency (EMA) 2019a). The Israeli Ministry of Health conducts a protocol for healthcare professionals, managers, and importers to carry out risk assessment to propose solutions implemented nationally if no generic alternatives are available (Schwartzberg et al., 2017). According to the NHS' Department of Health and Social Care, performing risk assessment for joint procurement is recommended to avoid local duplication (Root, 2018; Government of the United Kingdom Department of Health and Social Care, 2019a). The National Patient Safety Agency's (NPSA) rapid response report emphasizes the risk posed by a lack of medicine and its potential impact on patients. Here, risk factors particularly relate to a determined medicine-group that may be pre-defined according to treatment delay, facilitating the work of healthcare professionals, and equipping them with the necessary tools needed to prevent harm from delayed or omitted treatment (UK Medicines Information, 2010).

There is a growing awareness of risk-management strategies that prospectively assess risks from shortages by focusing on national-legislation and regulatory-framework mapping that involves “out-of-stock” situations (Acosta et al., 2019). However, addressing these risks does not require any assessment that may occur following or prior to substitution. To illustrate, a survey on clinical risk management (Manser et al., 2016) revealed a significant variability in the implementation of risk assessment methods where 43% and 31% of hospitals report no use of the FMEA or RCA in clinical risk management.

Given this variability, it follows that only 18 out of 34 participants of our survey reported being aware of the steps necessary to carry out a risk assessment. While 61.7% of participants in this study are aware of risk assessment applicable in medicine shortages, 44.1% apply it in everyday practice. According to the survey, participants from seven countries are aware of RCA to be used to mitigate shortages, while 14 participants from nine countries acknowledged FMEA/HFMEA as a risk assessment tool in shortages. It is then questionable if RCA is a tool to be implemented in risk assessment stemming from medicine shortages. Bonnabry et al. views RCA as a suitable methodology when incidence/errors are frequent; however, retrospective methods are not deemed suitable for low incidence or difficult to predict situations (Bonnabry et al., 2005). Assessing risks after a failure is entirely unacceptable since the patient's health outcomes may be detrimental (Bonnabry et al., 2005). Consequently, having a more prospective approach to manage high risks in healthcare processes, similar to the aviation industry, has received greater acknowledgement and has become more accepted among healthcare professionals since 2002 when the FMEA was transformed into the HFMEA (DeRosier et al., 2002; Bonnabry et al., 2005; Kurgat et al., 2019). The sterilization of surgical instruments and the intravenous administration of medicines already implemented these approaches (Wetterneck et al., 2004; Linkin et al., 2005; Kurgat et al., 2019). By proactively detecting shortage risks, health professionals may establish therapeutic alternatives in advance especially for critical medicines in order to allow healthcare providers to be ready if shortages occur. The survey results reflect these findings, where more participants (35.2%) opted for prospective compared to retrospective (26.4%) risk assessment.

Although slightly more than half of all participants were aware of risk assessment as a tool to mitigate medicine shortages, only 4 out of 17 applied it to hospital pharmacies. Bearing in mind that the majority of participants are hospital pharmacists, this result does reflect their particular outlook as healthcare professionals.

Only participants from five countries 5/34 (14.7%) stated that their respective legislation stipulating the implementation of risk assessment in medicine shortages has been put into practice. According to most participants (26/34—76.4%), there are no changes to legislation in their respective countries in the near future. Their responses point to a lack of the integration of risk assessment into mitigating shortages. Therefore, the creation of legislative tools facilitating healthcare professionals in establishing and meeting risk-assessment objectives is recommendable *via* a proactive, prospective and preventative manner, rather than a reactive and retrospective one (Clarkson, 2010).

In line with recommendations for managing shortages (Ordre des pharmaciens du quebec service des communications, 2012; Health Products Regulatory Authority (HPRA), 2018), 12/34 participants (35.3%) perform risk assessment in multidisciplinary teams, thereby promoting the equitable use of scarce resources and prioritizing treatments (Chiozza and Ponzetti, 2009; Health Products Regulatory Authority (HPRA), 2018). A majority of participants noted that nurses and pharmacist technicians are included as multidisciplinary team members. In Belgium, members may be chief medical or executive officers, as well as in Finland. Having executives on team provides support to the implementation of treatment plans and shortage-mitigation guiding procedures. A list of medicines detailing what consequences may occur if the medicine becomes unavailable is also crucial. The participants cite a list following a similar global pattern of frequent shortages described in Acosta et al. (2019).

Fifteen of 34 (44.1%) participants reported implementing risk assessment in their daily work, while 24/34 (70.6%) do not have risk assessment integrated within medicine-shortage mitigation protocols. As reported, the lack of integrated risk assessment confirms that risk assessment is implemented empirically, based on experience, knowledge and the competences of the healthcare professionals, rather than from following structured, defined procedures.

Already having risk assessment guidelines in place allows for healthcare professionals to take individual patient profiles into account, including a patient's clinical status and comorbidities. According to the ASHP, every healthcare setting should perform an impact analysis in order to assess the effect of a shortage on work processes in a hospital pharmacy and its consequences for clinical outcomes (Fox and McLaughlin, 2018). Patients must be under supervision so that alternative medicines may be introduced where appropriate. Risks from increased adverse effects and deteriorated health must be taken into account before deciding on a substitute medicine (Fox and McLaughlin, 2018; Root, 2018).

The participants' high response rate does reflect the continuing problems in medicine shortages where they pointed to potential health risks associated with substitution (particularly with a medicine's reconstitution, administration patterns, dosing, stability, and adverse events profiles). The participants also widely acknowledge incorporating risk assessment and how critical medicine is to developing medicine guidelines to reduce potential harm from an interrupted or delayed treatment. This is in concordance with the Royal Dutch Society of Pharmacists who provide pharmacist-gathered data and supply-chain updates on the Farmanco website to inform on a shortage's probable cause, its duration, and potential alternatives (The Royal Dutch Pharmacists Association (KNMP), 2019). Through disseminating and sharing information on the substitution of medicines, healthcare professionals are supported in shortages (The Royal Dutch Pharmacists Association (KNMP), 2019). Although Australia, Canada, and the US update their databases regularly, (Vida et al., 2016; Acosta et al., 2019), the majority of shortage-reporting systems in Europe do not do so frequently as well as do not appear to have information on possible substitutes and clinical guidance for healthcare professionals (European Medicines Agency (EMA), 2019a).

There is no report of the 2013 EMA guiding document describing resources for issuing treatment recommendations during shortages. Although this guiding document does not provide the means to use such recommendations on a patient-impact analysis for risk assessment, it does establish the foundation of a further document (issued 3 years later) describing criteria to establish and distinguish critical from non-critical medicine shortages based on patient-health consequences (European Medicines Agency (EMA), 2013). It is of concern that only one participant refers to these documents since it may suggest that healthcare professionals are not actively using or consulting them. The participants also made no reference to subsequent templates for risk indicators for shortages issued by the EMA in 2015 that focus on manufacturing and quality issues (European Medicines Agency (EMA), 2015). The EMA also issued two more documents in 2019 (outside the survey time) that notify on shortages and communication to the public (European Medicines Agency (EMA), 2019a; European Medicines Agency (EMA) 2019b). The communication document provides a list of databases on alternatives with accompanying details on their application in Denmark, Germany, and Norway, among others (European Medicines Agency (EMA), 2019a). Based on our findings, we are led to believe that the EMA should do more with its communication strategy since the participants reference none of these documents. Working in cooperation with healthcare professionals' associations across Europe could better serve to disseminate information and facilitate management of medicine shortages at all healthcare settings, as they would be the best channels to promote and inform on the aforementioned documents.

The survey results demonstrate wide variation concerning the time needed to manage a shortage *via* risk assessment, stretching from 1 to 20 h per week. The literature confirms this variation in Europe (Caulder et al., 2015; Pauwels et al., 2015; Miljkovic et al., 2019). Developing tailor-made measures to mitigate shortages does remain a challenge due to time concerns among some participants.

Sharing information is a cornerstone of the new strategy for combating shortages issued by the French Ministry of Health, which emphasizes that both patients and healthcare professionals have the right to transparent and timely information on medicine shortages (France Assos Santé, 2018). It is therefore of concern that risk-assessment based outputs are typically not shared with other key stakeholders participating in mitigation, as stated by 27/34 (79.4%) participants.

Although there are multi-stakeholder initiatives in place to improve shortage management *via* risk assessment, 23/34 (67.6%) participants make no mention of their application in Europe. Furthermore, since 30 (88.2%) participants reported not being aware of any published document/publication that describes how to reduce the impact of medicine shortages, initiatives intended for handling medicine shortages need better dissemination and application. These should not only be based on creating and enforcing databases for collecting information on medicines affected by shortages, but also apply approaches based on risk management tailorable to country specifics and prospectively address shortages (Acosta et al., 2019).

Numerous guidelines state that shortages are best handled prospectively (Institute for Safe Medication Practices Canada, 2006; Parenteral Drug Association (PDA), 2014; Fox and McLaughlin, 2018). Accordingly, 15 (44.1%) participants reported successfully mitigating medicine shortages through risk assessment, while 29/34 (85.3%) considered risk assessment useful for mitigating shortages. Ultimately, the majority of participants share the view that not exposing patients to harm stemming from medicine shortages is the main outcome of successfully-led mitigation. In order to assess if there may be any harm to patients originating from shortages, it is important to tailor tools that could assist in recording and sharing the impact of shortages as well to create treatment guidelines addressing shortages (Phuong et al., 2019). This includes potential replacement treatments when the need arises.

### Limitations of the Study

Although this study aimed at capturing a European perspective regarding risk assessment in medicines shortages, there are a number of limitations. It can be difficult to draw vigorous conclusions from research based on conducted surveys due to issues regarding the recruiting of participants and having a representative sample (Banerjee et al., 2014). Participants might also provide answers expected to be more acceptable, which may affect the validity of the research findings (Beiderbeck et al., 2004). The majority of answers also came from hospital pharmacists, which severely limits conclusions compared to wider healthcare and multi-stakeholder scales. Study participants also provided answers based on their own knowledge and experience. While these are supported by the literature and available guidelines for risk assessment, it still cannot eliminate all possible biases. Moreover, only COST Action 15105 members, hospital pharmacist EAHP members and their associated colleagues were involved in this study, while other healthcare professionals/stakeholders that might be involved in medicine shortage mitigation across Europe did not participate and provide their feedback in this study. Finally, although it would be of assistance to relate the type of risk detected with the specific medicines affected by a shortage among the participants, it was not included in the original survey as the purpose was to focus directly on risks detected emerging from risk assessment (see **Table 5**). Therefore, single medicines/substances are not able to be listed directly within this study by their International Nonproprietary Names. This limitation should be remedied in further studies.

## Conclusion and Recommendations

Our study points to a lack of systematically organized tools used to prospectively analyze clinical as well as operationalize risk stemming from medicine shortages in healthcare. Although, the awareness of prospective risk assessment does exist among healthcare professionals, there currently appear to be a lack of legal instruments and sufficient data confirming the necessity and usefulness of risk assessment in mitigating medicine shortages in Europe. As a result, it is recommendable that healthcare professionals be given the skills to perform risk assessment *via* established protocols integrated into their everyday work.

Risks originating from medicine substitution when shortages occur are the main reported concern of the majority of the participants. Through the prospective identification and description of potential risks, healthcare professionals may better account for and manage the likelihood and severity of a shortage's impact. Notwithstanding, the risk assessment outputs provided have typically been seen or not shared among all key stakeholders. In view of the discrepancy, it is suggested that multi-stakeholder communication is necessary in order to avoid duplication of efforts and to assure effectiveness of medicine-shortage mitigation measures. This should be a subject of future research.

The participants perform risk assessment equally for particular medicines, such as immunoglobulins, blood products, and those for oncology as well as for essential medicines such as antibiotics, oral anticoagulants, diuretics, and antihypertensives. Assessment therefore knows no difference when risks to patient health needs are evaluated and potential harm is prevented. However, without an integrated structure in the workplace, risk assessment does not achieve its full potential.

All key stakeholders involved in managing medicine shortages should consider combining their efforts through conducting proactive and prospective risk assessment in a harmonized manner to support patient harm reduction. It is particularly important in hospitals where there are manpower issues. Hospital pharmacies employing more staff do perform more risk assessment compared with pharmacies with fewer staff. For this reason, apart from lack of skills in conducting risk assessment, there is insufficient available staff who are able to carry out risk assessment, despite its positive impact on patient safety. This insufficiency of staff must be addressed going forward. Moreover, greater information technology support is needed in carrying out risk prevention strategies *via* developing and providing a list of agreed substitutable medicines during shortages across countries. Key stakeholders within hospitals may undertake list development as part of Drug and Therapeutic Committee activities aimed at better handling medicine shortages.

A prospective approach in risk assessment not only allows the risks to be handled systematically in a timely manner, but also provides and influences a safety culture within healthcare settings. Through empowering healthcare professionals as active participants and not a mere spectator of ongoing risks stemming from shortages, it is possible to provide a safer environment for patients across healthcare settings. Consequently, based on participant responses, healthcare professionals must also invest greater effort to reduce adverse events due to shortages.

## Data Availability Statement

The datasets generated for this study are available on request to the corresponding author.

## Author Contributions

NM designed and carried out the study, including the preparation of questionnaire. NM conducted the data management and performed analysis and interpretation of data needed for the manuscript together with MK. BG, BM, IH, and TB revised critically the questionnaire and contributed to critical analysis and interpretation of data. The following authors contributed in acquisition of data from respective countries: GG, MJ (Austria), TDR (Belgium), AC, GL, BT, LN, VV-A (Bosnia and Herzegovina), DK-P (Croatia), EK (Cyprus), HF, KS (Denmark), MS (Estonia), JL (Finland), GB, RH (France), TH-T (Germany), LT (Greece), LH, RV (Hungary), JP (Ireland), MD (Israel), PP (Italy), IS (Latvia), BA, ND-K (Montenegro), AnM (Norway), IA (Romania), DR, PZ (Serbia), FT (Slovenia), AlM, CG (Spain), HJ (Switzerland), and DP (United Kingdom). NM prepared the first draft of the manuscript and the literature review. All authors read, commented on and contributed to subsequent iterations of the manuscript for the accuracy of the content. All authors finally approved the version to be published and agreed to be accountable for all aspects of the work in terms of its accuracy and integrity. NM is the corresponding author.

## Funding

This research is based upon work from COST Action CA 15105 [European Medicines Shortages Research Network—addressing supply problems to patients (Medicines Shortages)], supported by COST (European Cooperation in Science and Technology), an EU-funded program. The funders had no role in study design, data collection and analysis, decision to publish, or preparation of the manuscript.

## Conflict of Interest

KS is employed by Nomeco A/S. HF is employed by Amgros I/S.

The remaining authors declare that the research was conducted in the absence of any commercial or financial relationships that could be construed as a potential conflict of interest.

## Footnotes

1 Other medicines mentioned in which a risk assessment is conducted include: digoxin, disulfiram, fluspirilene, neostigmine, remifentanil, sodiumchoride solution, tranexamic acid and warfarin.

2 The time invested in managing shortages, including risk assessment, is found to span from up to one or two hours per day in Romania and Greece to ten hours per week in Ireland.
