# Supplementary material for: Prospective Risk Assessment of Medicine Shortages in Europe and Israel: Findings and Implications
Source: Front Pharmacol. 2020 Mar 26;11:357. doi: 10.3389/fphar.2020.00357 (PMC7114887; doi:10.3389/fphar.2020.00357)
Supplement: Supplementary file 1 [file DataSheet_1.pdf]

Survey of Prospective Risk Assessment Practices in Medicine Shortages

Dear Sir/Madam,

On behalf of the COST Action 15105 Working Group 4, which is dedicated to the clinico-pharmacological needs of patients in medicine shortages, we would like to ask for a brief moment of your time in order to gather information on any possible insight into introducing alternative medicines and classifying the criticality of shortages. It need not matter whether you have been assessing this risk or that you are a healthcare professional yourself, as I value any input you may be able to give in relation to shortages in general.

Bearing this in mind, we would like to invite you to participate in the “Survey of Prospective Risk Assessment Practices in Medicine Shortages” aiming at identifying risk assessment procedures as a medicine-shortage mitigation strategy. Furthermore, this Survey will allow us to gain insight into the implementation of the aforementioned procedures in everyday practice and what their implications are regarding the medicine-shortage mitigation process.

Please read the questionnaire carefully and provide the most accurate answers in accordance with you daily practice, experience and available legislation acts, accompanied by relevant references. We highly appreciate your time and willingness to participate in this research project. For this reason, we would be extremely grateful if you were to accept the invitation to co-author the manuscript that will be prepared afterwards, aiming at publishing it in a peer-reviewed scientific journal.

We would also like to ask you to recommend and provide contact details by email of potential survey respondents from your professional network, whom you consider relevant for this research topic, and willing to contribute as co-authors in the expected publication.

Thank you for taking the time to read this information fully. Please complete the questionnaire by no later than July 1st, 2018. This survey should take no longer than 15 minutes to complete. If you have any questions regarding the survey and this study feel free to send an email to nenad.hedren@gmail.com or nenad.miljkovic@iohbb.edu.rs

Yours sincerely,

Nenad Miljković

Tomasz Bochenek

Isabelle Huys

Branislava Miljković

Untitled Section

EUROPEAN COOPERATION
IN SCIENCE AND TECHNOLOGY

Respondent's Contact Details

1. **Name and Surname**

2. **Occupation and Workplace**

3. **Country**

4. **Email Address**

Risk assessment as a medicine shortage mitigation strategy

5. **1.1 Are you aware of any risk assessment procedures that can be used to mitigate medicine shortages?**
   Mark only one oval.

   - Yes *Skip to question 6.*
   - No *Skip to question 7.*

Risk assessment as a medicine shortage mitigation strategy

6. **1.2 Please name the type of risk assessment that can be used to mitigate medicine shortages?**
   Mark only one oval.

   - Hazard Analysis and Critical Point (HACCP)
   - Root Cause Analysis (RCA)
   - Failure Mode and Effect Analysis (FMEA)
   - Healthcare Failure Mode and Effect Analysis (HFMEA)
   - Other:

Risk assessment as a medicine shortage mitigation strategy

7. **1.3 Please describe the steps of the risk assessment procedures you are familiar with?**

Risk assessment as a medicine shortage mitigation strategy

8. **1.4 Do you implement risk assessment in your daily work?**
Mark only one oval.

- ( ) Yes *Skip to question 9.*
- ( ) No *Skip to question 12.*

Risk assessment as a medicine shortage mitigation strategy

9. **1.5. Please choose one of the options listed below for the setting where the risk assessment takes place:**
Mark only one oval.

- ( ) hospital pharmacy
- ( ) community pharmacy
- ( ) health authority
- ( ) manufacturing facility
- ( ) wholesaler facility
- ( ) Other: ____________________

Risk assessment as a medicine shortage mitigation strategy

10. **1.6 Are you conducting the risk assessment within multidisciplinary teams?**
Mark only one oval.

- ( ) Yes *Skip to question 11.*
- ( ) No *Skip to question 12.*

Risk assessment as a medicine shortage mitigation strategy

11. **1.7 Please name the type of professions included in the multidisciplinary team.**

____

Risk assessment as a medicine shortage mitigation strategy

12. **1.8 For which particular medicine are you aware that risk assessment - as a medicine-shortage mitigation strategy - is being performed in your country, (please provide international and brand names of the medicine(s), if possible)?**

____
____
____
____
____

Risk assessment as a medicine shortage mitigation strategy

13. **1.9 Is there a defined act stipulating the implementation of risk assessment during medicine shortages embedded within legislation in your country?**
Mark only one oval.

- Yes *Skip to question 14.*
- No *Skip to question 15.*

Risk assessment as a medicine shortage mitigation strategy

14. **1.10 Please provide a reference to the relevant legal or organisational document in English or in your native language.**

Skip to question 17.

Risk assessment as a medicine shortage mitigation strategy

15. **1.11 Are you aware of any other legislation which is to be implemented in the near future in your country?**
Mark only one oval.

- Yes *Skip to question 16.*
- No *Skip to question 17.*

Risk assessment as a medicine shortage mitigation strategy

16. **1.12 Please provide a reference to the corresponding legal or organisational document in English or in your native language.**

Risk assessment as a medicine shortage mitigation strategy

17. **1.13 Are you able to say how long risk assessment related to medicine shortages has been implemented in your country?**
Mark only one oval.

- Yes *Skip to question 18.*
- No *Skip to question 19.*

Risk assessment as a medicine shortage mitigation strategy

18. **1.14 Please give details in general terms of years and/or months.**

Risk assessment as a medicine shortage mitigation strategy

19. **1.15 Are you aware of any initiative for the implementation of risk assessment related to medicine shortages expected to take place in the near future in your country?**
Mark only one oval.

- Yes *Skip to question 20.*
- No *Skip to question 21.*

Risk assessment as a medicine shortage mitigation strategy

20. **1.16 Please provide details if possible.**

Risk assessment as a medicine shortage mitigation strategy

21. **1.17 Are there any published, governmental or nongovernmental documents on applied risk assessment in your country?**
Mark only one oval.

- Yes *Skip to question 22.*
- No *Skip to question 23.*

Risk assessment as a medicine shortage mitigation strategy

22. **1.18 Please provide a reference to the corresponding documents in English or in your native language. Would you please also describe the context in which the law is used in practice?**

Risk assessment as a medicine shortage mitigation strategy

23. **1.19 Are there any published governmental or nongovernmental documents dedicated to studying ways to reduce the impact of medicine shortages? Specifically, any documents related to applied risk assessment measures as a mitigation strategy in your country?**
Mark only one oval.

- Yes *Skip to question 24.*
- No *Skip to question 25.*

Risk assessment as a medicine shortage mitigation strategy

24. **1.20 Please provide a reference to the corresponding documents in English or in your native language.Would you please also describe the context in which the law is used in practice?**

The structure and process of risk assessment procedures

25. **2.1 How much time, if at all, would you say that you need to perform risk assessment related to medicine shortages in your job?**

26. **2.2 How frequently do you update risk assessment procedures applied as a medicine shortage mitigation strategy (for example: monthly, quarterly, annually)?**

The structure and process of risk assessment procedures

27. **2.3 If you apply risk assessment procedures, could you please name the most frequently detected risks when using a medicine shortage mitigation strategy (for example: medicine supply options; medicine substitutions; unknown/not followed methods of use of an alternative medicine, lack of information technology infrastructure, etc.)?**

The structure and process of risk assessment procedures

28. **2.4 Are risk assessment procedures part of the medicine shortage protocols/mitigation strategy in your workplace?**

Mark only one oval.

- Yes
- No

The structure and process of risk assessment procedures

29. **2.5 When conducted, are you obligated to report on the results of the risk assessment to official institutions inside your country?**

Mark only one oval.

- Yes
- No *Skip to question 33.*

The structure and process of risk assessment procedures

30. **2.6 What is the name of the institution or organisation which is notified? Please also describe how you communicate/share the aforementioned information.**

The structure and process of risk assessment procedures

31. **2.7 Do you have dual-channel communication with them?**

Mark only one oval.

- Yes *Skip to question 32.*
- No *Skip to question 33.*

The structure and process of risk assessment procedures

32. **2.8 Could you provide details on how the communication system you use works (for example reporting to a health authority, national/private health insurance funds, etc.)?**

The implications of risk assessment procedures regarding the medicine shortage mitigation process

33. **3.1 When completed, do you share the outcomes of the performed risk assessment for a specific medicine with other stakeholders in your country (such as manufacturers, health authorities, national/private health insurance funds)?**

Mark only one oval.

- Yes
- No

The implications of risk assessment procedures regarding the medicine shortage mitigation process

34. **3.2 Do you perform risk assessment together with other medicine shortage mitigation strategies?**

Mark only one oval.

- Yes *Skip to question 35.*
- No *Skip to question 36.*

The implications of risk assessment procedures regarding the medicine shortage mitigation process

35. **3.3 Please describe this additional strategy?**

The implications of risk assessment procedures regarding the medicine shortage mitigation process

36. **3.4 According to your professional experience, have you been able to mitigate medicine shortages successfully with risk assessment procedures?**

Mark only one oval.

- Yes *Skip to question 37.*
- No *Skip to question 38.*

The implications of risk assessment procedures regarding the medicine shortage mitigation process

37. **3.5 Could you please provide details on what grounds/basis the mitigation strategy was deemed to be successful?**

The implications of risk assessment procedures regarding the medicine shortage mitigation process

38. **3.6 Do you keep records on how quickly you can manage risk assessment when a medicine shortage abruptly occurs?**
Mark only one oval.

- Yes *Skip to question 39.*
- No *Skip to question 40.*

The implications of risk assessment procedures regarding the medicine shortage mitigation process

39. **3.7 Please provide details on the time needed to perform risk assessment in such situations.**

The implications of risk assessment procedures regarding the medicine shortage mitigation process

40. **3.8 Has there ever been a situation where the risk assessment procedure for a medicine shortage suggested a different mitigation strategy compared to the procedure with no risk assessment?**
Mark only one oval.

- Yes *Skip to question 41.*
- No *Skip to question 42.*

The implications of risk assessment procedures regarding the medicine shortage mitigation process

41. **3.9 Please provide details if possible.**

The implications of risk assessment procedures regarding the medicine shortage mitigation process

42. **3.10 How many times did you perform risk assessment for medicine shortages last year in your job?**

43. **3.11 Do you find risk assessment useful as a medicine shortage mitigation strategy?**

Mark only one oval.

- ( ) Yes
- ( ) No

Powered by Google Forms
